# Military Graveyards and Epitaphs in India—V

**Published:** 1889-03-30

**Authors:** 


### MILITARY GRAVEYARDS AND EPITAPHS IN INDIA.—V.

By an Old Indian.

(*Concluded from page* 401.)

The graveyard at Serinuggur Cashmere—or, as these words are now rendered, Srinuggur Kashmir—was, when visited by me a few days after the inspection referred to above, "a small railed enclosure in the Residency compound, which contained some eleven graves, and was, considering the general surroundings and locality, in a very creditable state of preservation and repair." It was, indeed, undergoing repairs at the time that I inspected it, while the Residency has since been removed higher up the stream towards the Tukth—I—Suleiman, and this being so, I need not say much about it. Moreover, it has, I believe, been since entirely re-cast or renovated. The graves were, of course, those of Europeans; for cremation is here as elsewhere the order of the day with Hindus, while the Mohammedans bury their dead apart, and only a few of them exhibited epitaphs.

One of these simply recorded the fact that a Captain Edward Shawe Powys, of the 61st Regiment, died at Srinuggur on the 23rd of September, 1855; while the death of a Dr. Reay, whose remains repose hard by, is ascribed to an avalanche in the neighbouring hills. I was subsequently shown a memorial window to him in one of the hovels at the wretched village of Pahilgam, in the Liddur Valley; while there is a small obelisk near the Sacred Cave of Ummernath to the memory of an officer who perished in that neighbourhood from, I believe, a similar cause. This was, at the time here indicated, the highest pillar of its kind, both as regards altitude or latitude in our dominions in the East; but one has since been erected to the memory of Dr. Stolicksa, near Leh, which exceeds it in both these respects, and what struck me most at this visit was the entire absence of all allusion to or commemoration of those officers and others who lost their lives by falling over precipices in the pursuit of game in the neighbouring mountains, or who succumbed to the attacks of wounded bears or other wild animals.

Returning, viâ Murree, from the so-called Happy Valley, I revisited the two burial-grounds of that place. I found nothing particular in the way of epitaphs in either; but the little mounds that indicated the last resting-places of the many children who had passed away in this place or its vicinity, "ere sin could blight or sorrow fade" their tender hearts, renewed in me a conviction I had already arrived at as to the greater prevalence of marasmus in this station than in any other with which I was acquainted. However that may be—and this is clearly not the place for a discussion on such a point—one of the cemeteries here referred to, or that nearest to the Mall between the Club and Presbyterian Chapel, is now, I think, disused; while the other (lower down) contained no inscription on the occasion of my visit that calls for notice here. This being so, and finding that a very similar description will suffice for the kindred-quoad locality, cemeteries of Mussoorie and Naini Tal, I will include them all in this paragraph.

As Mussoorie is one of the oldest hill sanitaria in India, while its central position or less conventional requirements have ever attracted to it a larger number of the Eurasian "upper crust," as well as of the Vieux Militaire or "Quay Hai" class than resorts to either or both of its fellows, so is its graveyard more freely studded with names of this kind than is the case elsewhere. Conspicuous among these was the name of the late General Colin Troup, C.B., with whom I was myself slightly acquainted, and who is otherwise so well known as to dispense me from the necessity of saying any more about him in this place. The cemetery at Naini Tal—which though in a smaller station than either of the above, yet occupies a more level area—contains, if I except a few inflated quasi-Biblical panegyrics on American missionaries, nothing strange or striking. But the recent terrible cataclysm will probably enhance its interest in the future, and the cemeteries at Kussowlee and Subathoo are so modern or circumscribed as to require no special description in this place.

The military cemeteries visited during my last tour of duty in the East partake so much of the general characteristics noted above as to almost dispense me from the necessity of dealing with them at all. The inquiry would, however, be incomplete without some allusion to these, and so taking a few, such as those of Meerut, Agra, Fyzabad, and Sitapur as specimens or representatives of the others, I will dismiss the subject for the present. A word or two must suffice for each of these, and John Lang's general description of an Indian burial-ground is so pertinent to all as to save me the trouble of describing any of them in detail. Alluding particularly to the cemetery of the first-named place—at which, as all old Indians know, he edited for many years the now defunct *Mofussilite* newspaper, he says that "an Indian churchyard presents a very different aspect to a churchyard in England or elsewhere. The tombs for the most part are very much larger. When first erected or newly done up they are as white as snow, formed, as they are generally, of chinam (plaster), which somewhat resembles Roman cement; but after exposure to one rainy season and one hot weather, they become begrimed and almost black. The birds flying from structure to structure carry with them the seeds of various plants and herbs, and these, if not speedily removed, take root and grow apace."

"A stranger," he continues, "wandering in the churchyard of Meerut, might fancy that he is amidst ruins of stupendous antiquity, if he were not aware of the fact that fifty years have scarcely elapsed since the first Christian corpse was deposited within those walls, which now encircle some five acres of ground, literally covered with tombs in every stage of preservation and decay,"* and the graveyards of India, he

---

* I reproduce here, lest this description may be considered as in any way exaggerated, a notice of another large graveyard elsewhere that appeared about this time from the pen of one of its correspondeuts in the pages of the *Delhi Gazette*. It runs as follows: "If there is one thing more than another that should remind us how transient our sojourn in this country is, that we have no abiding city here, it is a visit to the station graveyard. A sight more dreary, a scene more calculated to depress the spirit of the lonely visitor, it is difficult to imagine. If King Solomon in his wealth and wisdom could exclaim that all was vanity in this world, how much more should this desolation; the gloomy resting places of all that here were young and fair, or in this world gained greatness and renown; the neglected grave of the maiden, once so beautiful, so gay, adored as very goddess, now mouldering away beneath yon brittle slab, her name itself unknown, effaced by but a few seasons' sun and rain; the dismal spots where lie the Captain, brave in battle, or the statesman, wise in council; the monuments erected to their memories by admiring friends or sorrowing relatives now lying broken amongst the noxious plants that seem to feed on their corpses; the simple stone beneath which slumbers the child, the tender bud withered away in the fiery blasts of the Indian plains, cut off before it blossomed, the stone all but disappeared amongst the luxuriant grass, the inscription obliterated by mud and dirt; the brick and mortar tomb falling fast into ruins, with its glass covered tablet stolen or wantonly destroyed long since; the heathenish urn that once surmounted the column at whose foot it now lies to be carted off with the next removal of the rubbish; the unmeaning temples with roots falling in and pillars tumbling down; the mosque-like structures over the coffin of some bold adventurer the hiding-place of bats by night, and the cook-house of the grave-yard *chowkeedar* by day; the forlorn walks and the rank weeds of this jungle—how much more should the sights of this untimely ruin, early decay, and sad neglect bring home in its full force to the beholder the truth of the wise King's saying!" And after dwelling more particularly on the old cemeteries at Dinapore, which are, he continues, "nothing but fields of broken stones . . . . . heaps of nondescript masonry, a conglomeration of mortar, mud, and bricks, the like of which it would be difficult to find" he adds: "I believe amongst the whole of the tombs in these two cemeteries there are not a dozen to be found of solid stone, the remainder being what your correspondent strikingly calls 'cairns,' formed of bricks and mortar, the latter crumbled away long ago through the action of the atmosphere, while the former are just commencing to undergo the process of dissolution, of an inscription remaining in most instances not might have added, tell tales which he who runs may read, and be no worse, in either his civil or military capacity for the lesson into the bargain.

But this is not the place for such reflections, and the object that most attracted my attention on the occasion of my last visit to this place was a monument that was recently erected by the officers and men of the Buffs to the comrades they had lost by cholera, etc., in, I believe, one disastrous season in that not usually unhealthy station. The epitaph it bears is too long for transcription, but it records the names of two officers, three colour-sergeants, three company sergeants, six corporals, fourteen drummers, one hundred and eleven privates, sixteen women, and forty-three children; and if this butcher's bill is not big enough for your conception of the consequences of a visitation of cholera, you can turn to either side and satisfy yourself by further, and it may be fuller, evidence of its virulence and power. *Si quaeris monumentum circumspice;* and had I been questioned on the spot as to the antiquity of this enclosure I might have answered, like Mr. Lang's "stranger," that it had come down to us in its present shape from the days of Prester John or the Great Mogul.

The old graveyard that lies in the civil lines near the Capuchin Convent at Agra would require a volume to itself, so historic are its memories and so diversified its contents. Among these are the remains of that infamous Alsatian, Walter Rheinhardt, who raised himself from the humblest position to the steps of a throne. Nick-named Sombre, on account of his swarthy complexion, by his comrades, and thence called Sumroo by the natives, he instigated, if he did not actually superintend, the massacre of Patua, and he was indirectly the originator of the notorious Dyce Sombre case. But his career, though chequered enough to form the groundwork of a tragedy, does not concern us further here, and I introduce it only out of regard to the subject in hand. Hard by are the tombs of those Portuguese adventurers and Jesuits who, attracted to the court of Shah-Jehan by the scepticism or insouciance of his heir-apparent, the Prince Dara Shekoh, looked forward confidently to his conversion. But the triumph of his younger brother, Aurungzeb, blasted all these ambitious hopes, and for the rest the cemetery here referred to embraces a period and a history that are entirely foreign to the object of this inquiry.

Reverting thence to our original topic, let us visit the military burial-ground and see what that is like. Situated on the open plain, that serves for a parade-ground for all the troops of this garrison, in rear of the military "lines," it exhibits the usual dreary outline of such a place in the parched plains of Hindostan. That is, it is enclosed between four low walls, within which a few scraggy shrubs take the place of trees, and it is laid out in squares after the usual standard plan. With this difference, however, that the sectarian boundaries, prescribed by regulation or private compact between the chaplains of the two leading communions, are here supplemented by a third subdivision for the use of the local Baptist denomination. It contains, considering its size and other surroundings, fewer sepulchral mounds or large tombs than any other of the larger enclosures adverted to within, and these were quite devoid of interest to me.

---

even a trace. It is a dismal sight indeed to behold these columns, pyramids and pillars leaning over as if mourning themselves for those whose memory they were destined to hon ur; to see large masses of bricks sink deeper and deeper into the ground, crush under their weight the mouldering bones that lie in the decayed coffin; it is a dull, sorrowful walk amongst this lanky grass, this rank vegetation, and these dilapidated monuments; a cheerless place in which to hold communion with the souls that went before us, to raise our prayers to heaven in unison with the chorals of the sphere; a dark, unhappy spot, where mortals weep, and nature wears her mourning-cloak: it is a place, indeed, which should remind us that we are but strangers in this land, who once laid low have no loving parents, no sorrowing kinsmen to tend the sod that covers the departed, whose conventional tomb, erected at the lea t possible expense, once finished is left to take care of itself, and no more inquired after by man"

A very similar remark may, indeed, apply to the whole place, and the following are the only epitaphs that struck me as being, at the time here referred to, at all worthy of transcription. In one of these a wife exhorts her disconsolate husband to hope and resignation after this fashion—

> Weep not for me my Partner dear,
> Or shake at Death's alarm,
> 'Tis but the voice that Jesus sends,
> To call me to His arms.

While in the other a husband indulges his grief over the loss of his wife and children in a strain of fervour and a turn of phraseology that admit of no doubt as to his nationality and creed.

> I H S
> Sacred
> To the Memory
> of
> Marria Condon, the
> Beloved Wife of D. Condon,
> 41st Regt, Who Died At Agra,
> on 13 January 68
> Age 37 years
> And Also Her 2 Children Wilm
> And Arite. This stone is erected
> By Her Beloved Husband Who
> Sorly regrets
> their loss May their Soles
> rest in peace amen."

A very few lines must suffice for the cemeteries of Fyzabad and Sitapur, and I include the latter in this description solely on the ground that it was the last of its kind I visited in India. They are both fairly kept, and conveniently situated near the European Infantry quarter and civil lines, and the absence of all record in either of those who perished at these places, before or during the Mutiny, impressed me very unfavourably. At least I looked in vain for any such commemorative notification, but it may exist elsewhere, though of this I am doubtful. I know, however, that the names of these unfortunate victims of a bad cause are to be found in the pages of Kaye and Malleson, and for the rest I found ample evidence of the uncertainty of European life in that treacherous climate, in both.

Thus a column that was erected in the former, to commemorate the deaths that occurred during its tour of service in that country, of No. 1 Battery, 11th Brigade R. A. contained the names of eight officers—two of whom were doctors—and forty-four men; while another hard by enunciated the fact that the equally small force that succeeded it lost in that station alone from '71 to '73 inclusive, two non-commissioned officers and twelve men. Similarly the battery that succeeded this lost, during the year, from '73 to '75, that it passed at this place, three non-commissioned officers and seven drivers, and a tablet in one of the walls gave the names of the nine sergeants of Hill's 26th Cameronians, who died in India between October 1865, and December 1872. Why the other losses of this corps are ignored it is not for me to inquire; they are doubtless treasured up in more enduring memorials elsewhere, and it should not be forgotten in this connexion, that many regiments reserve details of this kihd for their county churches, or other national or affiliated institutions.

---

One may be a good physician, a good governor, a good grammarian; but without virtue one cannot be a good man. It is not the matter, but the virtue that makes the action good or ill; and he that is led in triumph may be yet greater than his conqueror. When we come once to value our flesh above our honesty we are lost.—*Seneca.*

